# Arene activation via π-bond localization: concepts and opportunities

**DOI:** 10.3762/bjoc.22.19

**Published:** 2026-02-09

**Authors:** Paul Meiners, Julian J Melder, Tobias Morack

**Affiliations:** 1 Organisch-Chemisches Institut, Universität Heidelberg, Im Neuenheimer Feld 270, 69120 Heidelberg, Germany

**Keywords:** arene activation, arene functionalization, dearomatization, metal–arene π-complexes, strained molecules

## Abstract

Dearomatization reactions of aromatic feedstocks constitute a highly efficient and conceptually powerful class of transformations for the synthesis of complex, three-dimensional molecular architectures with tailored physicochemical properties. Despite notable advances in dearomative methodologies over the past decades, the selective and controlled disruption of the aromatic core continues to represent a fundamental challenge in synthetic chemistry. In this review, we delineate the potential of π-bond localization within the aromatic framework as a general strategy for arene activation and dearomatization. Four distinct approaches are discussed, encompassing localization of the arene π-bonds through small-ring annelation as well as transition metal coordination to aromatic fragments in an η^2^-, η^3^-, and η^4^-fashion. The structural and reactivity consequences of these perturbations are analyzed in detail, and representative examples of stoichiometric and, where available, catalytic applications in synthesis are highlighted. Collectively, these concepts create a roadmap for the development of new strategies that harness π-bond localization to expand the synthetic utility of aromatic compounds.

## Introduction

**Figure 1:**
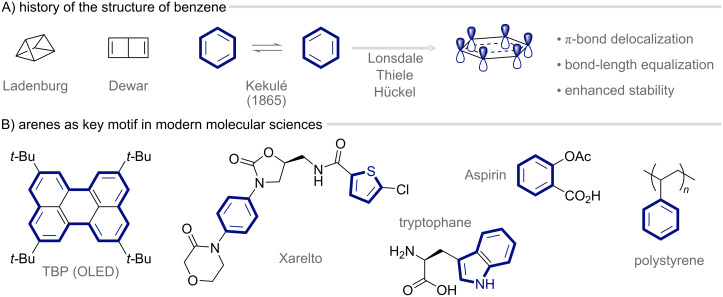
Aromatic molecules as the foundation of modern molecular chemistry.

Aromaticity has intrigued chemists since Michael Faraday’s isolation of benzene in 1825, marking the beginning of a concept that has shaped much of modern chemical thinking [1–2]. Despite its fundamental importance, aromaticity remains an abstract notion [3]. It is central to understanding molecular structure and reactivity, yet it cannot be measured directly [4]. This inherent elusiveness contributed to decades of debate over the structure of benzene, the prototypical aromatic molecule, until August Kekulé proposed his venerable representation: a six-membered carbon ring with alternating single and double bonds (Figure 1A) [5]. However, despite its strength in representing a planar cyclic arrangement of tetravalent carbon atoms, this formalism fails to accurately depict the observed reactivity, structure, and stability of benzene. It implies three rapidly equilibrating, localized double bonds, which conflicts with experimental observations.

Thiele’s introduction of partial valency [6], corroborated by Lonsdale’s groundbreaking structural studies [7], and Hückel’s application of molecular orbital theory [8] subsequently began to rationalize these observations and the unique chemical behavior of aromatic compounds, finally establishing the concept of aromaticity. This behavior manifests itself in numerous illustrative criteria, such as bond-length equalization, enhanced stability, and distinct magnetic and spectroscopic properties, none of which are entirely free of ambiguity [3].

Today, aromatic compounds are essential in both academic and industrial chemical applications (Figure 1B) [9–10]. Their ubiquity is the result of a decades-spanning effort devoted to leveraging aromatic stability in the development of methods that functionalize the periphery of arenes without disrupting the aromatic core, such as electrophilic and nucleophilic aromatic substitutions, transition metal-catalyzed cross-couplings [11], and C–H functionalizations [12]. Taken together with their natural abundance, these transformations have established arenes and heteroarenes as versatile synthetic building blocks in pharmaceuticals, agrochemicals, and materials science (Figure 1B).

**Figure 2:**
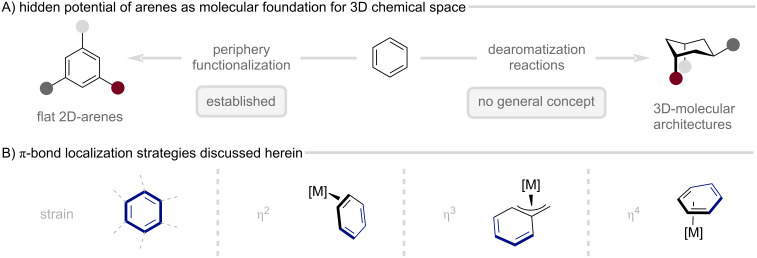
Arenes as springboards to three-dimensional chemical space and strategies toward arene activation via π-bond localization discussed in this review.

In contrast, direct modification of the central aromatic core, i.e., controlled disruption of aromaticity, remains a fundamental challenge in synthetic chemistry and leaves the vast potential of planar arenes as springboards to three-dimensional molecular architectures largely untapped (Figure 2A) [13]. Enforcing localization of the arene π-system achieves precisely this: it activates the arene through induction of an alkene-like character, thereby presenting a twofold opportunity: (1) to advance dearomative chemistry into a general, efficient synthetic strategy that enables an entirely new retrosynthetic logic, and (2) to enable previously inaccessible mechanistic pathways for selective arene functionalization.

With this review, we aim to assess the current landscape of synthetically applied systems that achieve arene activation through π-bond localization (Figure 2B). While most examples remain limited to stoichiometric studies probing the structural and reactivity consequences of perturbing aromatic stability, they collectively unveil a rich and largely untapped conceptual space that continues to simmer beneath the surface of the literature, holding remarkable promise for translation into catalysis.

In the first chapter we examine strain-induced π-bond localization for arene activation, following the historical traces of the Mills–Nixon hypothesis, its fallacies and the implications of strain on arene structure and reactivity. The following chapters introduce the broader mechanistic framework of transition metal coordination to a subset of the aromatic system, specifically η^2^-, η^3^-, and η^4^-coordination. These partial coordination modes effectively clamp the otherwise delocalized π-system, disrupting aromaticity and unveiling the latent double-bond character within the aromatic ring. This dramatically increases reactivity, enabling alkene-like transformations with aromatic molecules that are inaccessible by conventional synthetic methods, thereby complementing traditional arene chemistry. Other modes of activation, such as the η^6^-coordination of arenes by π-Lewis acids, are specifically not included in this classification as the π-bonds remain fully delocalized and these modes have been extensively reviewed elsewhere [14–15].

## Review

### Small-ring strain-induced arene activation

#### Historical context

**Figure 3:**
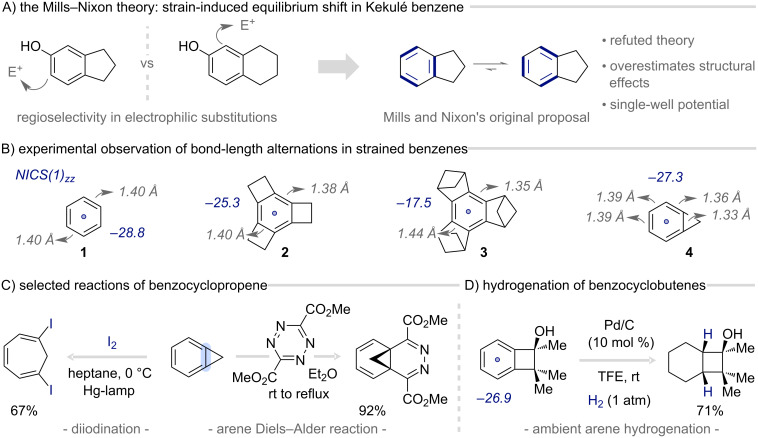
Structure and synthetic utilization of strained arenes; NICS: nucleus independent chemical shifts [26–28].

The concept of π-bond localization in arenes and its influence on reactivity can be traced back to the early 1930s, when Mills and Nixon investigated how the annelation of small rings to aromatic systems influences the regioselectivity of electrophilic substitutions in tetralin vs indane (Figure 3A) [16]. Building on Kekulé’s early oscillation model for benzene, they proposed that small-ring annelation perturbs the equilibrium between the two assumed cyclohexatriene structures, shifting it to one side [17]. Although this original hypothesis was incorrect, its implications have been the topic of longstanding academic debate and have inspired numerous structural studies [18–21]. These later investigations demonstrated that arenes fused to highly strained frameworks (such as Bürgi’s tris(bicyclo[2.1.1]hexeno)benzene (**3**)) do indeed exhibit considerable deviations from uniform bond lengths [22], despite being much more subtle than predicted by Mills and Nixon’s theory, and for different reasons (Figure 3B) [23]. Importantly, aromaticity persists globally as the π-system can remain largely delocalized (compare nucleus independent chemical shift (NICS) values, Figure 3). Instead, the σ-framework distortion enforces an alkene-like double-bond character at the junction, accompanied by enhanced reactivity unusual for aromatic systems [24–25]. In contrast to the breadth of structural and theoretical studies, synthetic utilization remains underexplored, despite the growing evidence that strain-induced arene activation offers powerful opportunities in reactivity design. Notably, the small-ring annelation exerts a dual influence: it lowers kinetic barriers and enhances regioselectivity, with strain-release providing the key thermodynamic driving force to overcome aromatic stabilization.

#### Synthetic applications

One of the earliest demonstrations of the unusual reactivity of small-ring-annelated arenes is found in benzocyclopropene, which undergoes facile dearomatization [29]. For example, upon exposure to iodine and UV-light, it transforms into 1,6-diiodocycloheptatriene, highlighting the pronounced double-bond character of the benzocyclopropene framework [30–31]. The reaction likely operates through an initial dearomative radical diiodination followed by a norcaradiene–cycloheptatriene rearrangement. Leveraging this enhanced reactivity, benzocyclopropene engages in inverse-electron-demand Diels–Alder reactions with several electron-deficient dienes, furnishing distinctive methano-bridged structures (Figure 3C) [32–35]. Similarly, small cyclophanes are known to act as reactive dienes in Diels–Alder chemistry [36–37]. More recently, Lu and co-workers reported the unusually facile hydrogenation of the aromatic core of highly substituted benzocyclobutenes using simple Pd/C under ambient conditions [38]. The pronounced substituent effects on the hydrogenation rate highlight the key role of small-ring strain in this generally challenging transformation. Complementary to these strictly strain-driven examples, Vollhardt’s studies on phenylenes show that enhanced reactivity can also derive from a combination of structural and electronic effects. Fusing cyclobutadiene motifs to benzene perturbs its π-system by introducing local antiaromatic character that, together with ring strain, increases π-bond localization, rendering phenylenes more susceptible to hydrogenation, metal complexation, ring opening, and cycloaddition reactions [39].

Building on these intriguing studies and the ongoing renaissance of strain-release-driven catalysis [40], harnessing small-ring strain to drive dearomatization is poised to open the door to new avenues of synthetic discovery.

However, unlocking the full potential of arene π-bond localization as a synthetic strategy is contingent on its successful translation into a catalytic framework, which fundamentally requires the activation to be external. The following sections therefore focus on the selective disruption of aromaticity through the formation of transition metal–arene π-complexes. Transient and reversible coordination of transition metals to aromatic π-systems is a key feature of many catalytic transformations, with the oxidative addition of palladium to aryl halides serving as a prominent example [41]. In addition to such fleeting intermediates, recent decades have seen the emergence of numerous well-defined metal–arene complexes, sufficiently stable to enable systematic exploration of their rich and versatile organic chemistry.

### η^2^-Coordination-based dearomatization agents

**Figure 4:**
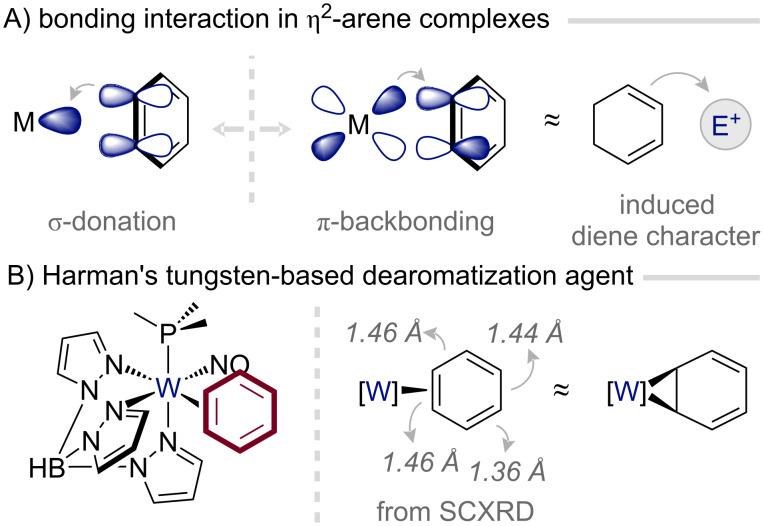
Bonding and reactivity of η^2^-coordinated aromatic systems [44,46].

Among the numerous ways in which transition metals can engage with a subset of the arene π-system, selective η^2^-coordination by electron-rich fragments stands out as the most well-defined and widely exploited mode (Figure 4) [42]. The stability of such η^2^-arene complexes rests on a finely balanced synergistic interaction: the metal center accepts electron density from a filled π orbital of the arene while engaging in π-backbonding, donating electron-density into an empty π* orbital (Figure 4A) [43]. This interaction not only stabilizes the metal–arene bond but also profoundly influences the electronic character of the aromatic ring. The ring becomes more electron-rich, akin to substitution with electron-donating groups, and exhibits distinct distortions in the bond lengths of the ring, consistent with localization of the π-system (i.e., dearomatization; Figure 4B) [44–46]. Collectively, these changes alter the innate reactivity of the arene, steering η^2^-coordinated systems toward electrophilic addition, cycloaddition, and hydrogenation processes.

**Figure 5:**
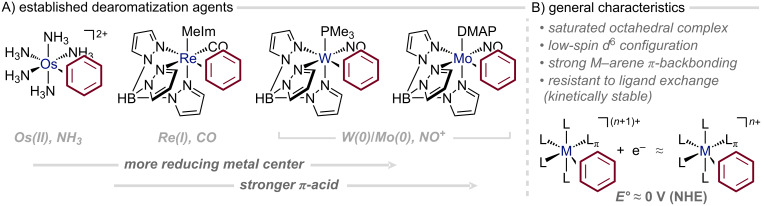
Illustrative selection of η^2^-coordinating dearomatization agents; MeIm: *N*-methylimidazole, NHE: normal hydrogen electrode.

While many transition metals can engage aromatic rings through η^2^-coordination, only a handful have proven synthetically valuable for transforming the bound arene [47]. Over recent decades, a distinct class of such η^2^-coordinating dearomatization agents has emerged: saturated octahedral complexes that feature pronounced metal–arene π-backbonding. A landmark in this field was published in 1987, when Taube reported the now famous pentaammineosmium(II) η^2^-benzene complex: the first system to yield kinetically stable η^2^-arene complexes that resist ligand exchange at room temperature in solution (Figure 5) [48]. This breakthrough opened the door to the isolation of such complexes and their subsequent exposure to reaction conditions that more labile systems could not tolerate. Crucially, most reagents used in organic synthesis react preferentially with the arene ligand rather than the metal center [45].

Building on the electronic blueprint established by the osmium system, Harman introduced a new generation of dearomatization agents based on Re(I), W(0), and Mo(0), tailoring the redox potentials of tris(pyrazolyl)borate (Tp)-based fragments to emulate that of the parent {Os(NH_3_)_5_}^2+^ fragment (Figure 5) [46,49]. Progressing down the series from Os(II) to W(0), the increasing π-backbonding strength that accompanies lower metal oxidation states translates directly into heightened reactivity of the η^2^-bound arene. These successors not only surpass the Os(II) system in their ability to activate coordinated arenes, unveiling previously inaccessible reaction pathways, but also introduce an additional dimension of control: chirality at the metal center. The resulting enantioenriched complexes have enabled a suite of asymmetric transformations (vide infra) [47].

#### Structure and properties

**Figure 6:**
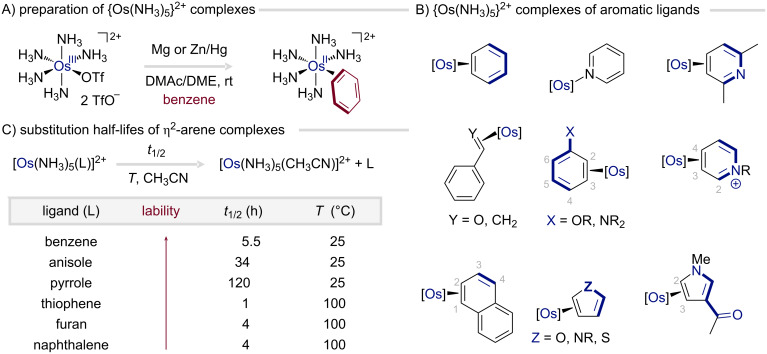
Preparation, lability and most stable linkage isomers of pentaammineosmium(II) complexes.

To rationalize and predict the enhanced reactivity of η^2^-bound arenes, it is instructive to first examine the properties of the archetypal pentaammineosmium(II) system prior to discussing synthetic utility (Figure 6). The five ammine ligands, strong σ-donors with negligible π-interaction, combined with the d^6^ configuration render the Os(II) center highly electron-rich and an exceptional π-base [43]. This electronic configuration promotes strong binding to π-acidic ligands, effectively compensating for the loss of aromaticity upon η^2^-coordination to arenes, while disfavoring oxidative addition due to geometric constraints of the octahedral complex. The resulting η^2^-arene complexes, encompassing both arenes and heteroarenes, display remarkable substitution inertness, even toward strong π-acids and good σ-donors. The substitution rate being relatively independent of the incoming ligand is consistent with a dissociative substitution mechanism. Typically, coordination occurs at positions that minimally disrupt the arene π-system (Figure 6C), so suitable aromatic ligands are limited to those lacking strongly π-acidic functional groups, e.g., alkenes, alkynes, aldehydes, certain ketones, and nitriles. Lewis-basic heteroarenes, such as pyridines or imidazoles, tend to coordinate via nitrogen instead, disfavoring η^2^-binding (Figure 6B) [45].

#### Application in organic synthesis

**Scheme 1:**
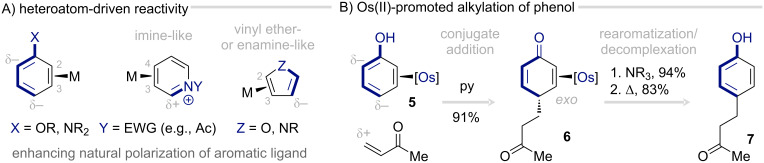
Heteroatom-directed reactions of η^2^-arene complexes [45,50].

Organic transformations of η^2^-coordinated arenes can be broadly divided into three categories, depending on what dictates reactivity: the polarization of the free molecule, the site of metal coordination, or the backbonding interaction itself. Early studies emphasized heteroatom-driven reactions in which metal coordination enhances the natural polarization of the molecule, primarily by disrupting its aromaticity. For example, a π-base preferentially binds to the C(2) and C(3) positions of monosubstituted arenes, maintaining linear conjugation between the substituent and the unbound portion of the aromatic ring (Scheme 1A). Thus, coordinating the metal to a benzene ring with a single π-donor substituent increases the interaction between the donor group and the uncoordinated diene moiety, further activating the uncoordinated *ortho* and *para* positions for electrophilic addition. Comparable effects are observed for furans and pyridines, where η^2^-coordination accentuates their vinyl ether and imine character, respectively.

As an illustrative example, treatment of the phenol complex **5** with methyl vinyl ketone and pyridine affords 4-alkylated 4*H*-phenol complex **6** as the conjugate addition product [50]. The initial addition occurs on the side of the phenol ring opposite (*exo*) the coordinated metal fragment. Subsequent exposure to a moderate base induces rearomatization. Upon heating, the raspberry ketone **7** is released in high yield (Scheme 1B).

**Figure 7:**
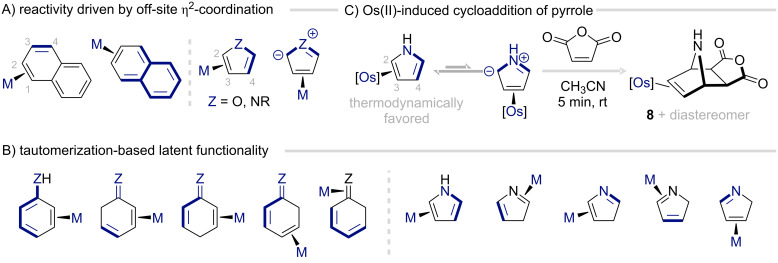
Latent functionality through transient metal binding.

The second class of reactivity emerges when the metal binds the arene at a site other than its thermodynamically preferred position, giving rise to fleeting but highly reactive intermediates (Figure 7). Such species are typically inferred from the product rather than observed directly. A defining feature of η^2^-coordinated aromatic complexes is their fluxionality [47]. Despite clear thermodynamic preferences for specific binding sites, the metal can migrate across the π-system without detaching from the aromatic ring (Figure 7A). This flexibility allows for the generation of multiple organic functionalities from a single class of aromatic molecules, depending on the coordination site. When using arenes with the ability to tautomerize, additional latent functionality is introduced (Figure 7B). This dynamic binding behavior provides greater overall versatility, but precise control of the metal binding site is critical to its utility.

For example, while Os(II) displays a marked preference for coordination at the C(2) and C(3) positions of pyrrole, it can also access the less stable 3,4-η^2^-isomer (Figure 7C) [51]. This species exhibits significantly higher reactivity in certain transformations. In this way, η^2^-coordination of pyrrole transforms the aromatic framework into an azomethine ylide intermediate. This reactive species can engage in a [3 + 2] cycloaddition with activated olefins such as maleic anhydride to form 7-azabicycloheptene complex **8** rather than an electrophilic addition product. An analogous scenario unfolds in η^2^-naphthalene complexes, where transient formation of the less favored 2,3-η^2^-isomer imparts *o*-quinodimethane character (Figure 7) [52].

**Figure 8:**
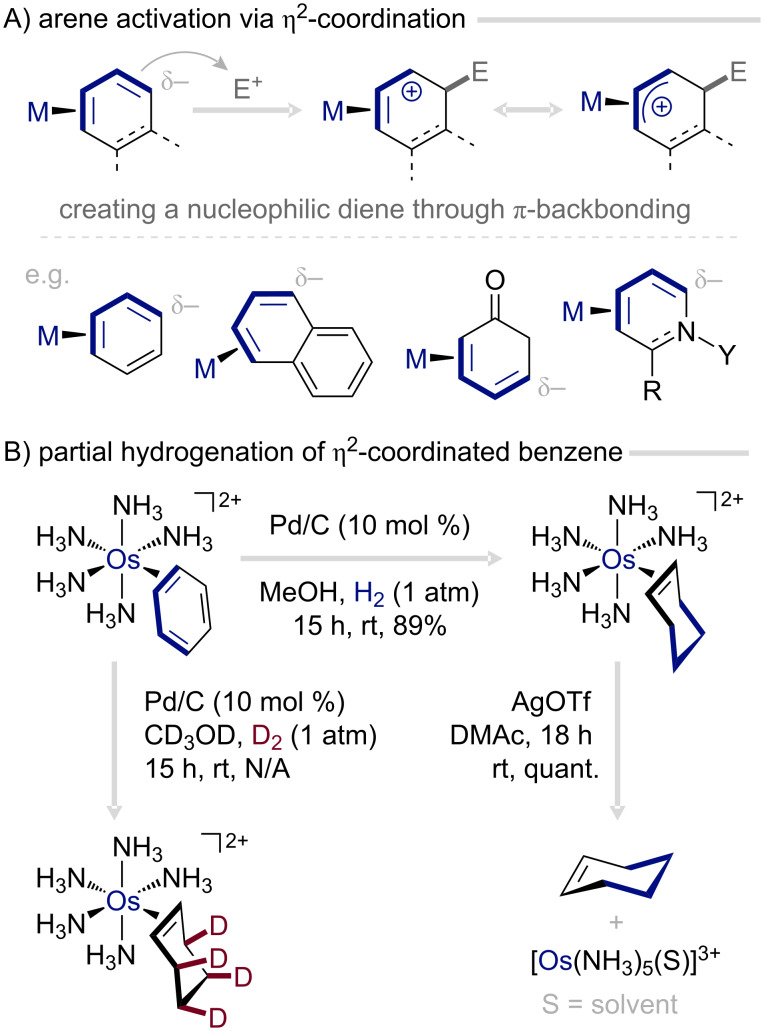
Selective hydrogenation of η^2^-coordinated benzene to cyclohexene under ambient conditions [53–54].

The third reactivity mode is purely activating in nature, with the metal acting as a π-donor that increases and localizes the electron density of the aromatic π-system. Such binding imparts a conjugated diene character onto the uncoordinated portion of the arene (Figure 8A). In contrast to the earlier reaction modes, the aromatic system lacks substituents or other pre-existing functionalities that would steer its reactivity. The hydrogenation of η^2^-arene complexes illustrates this perfectly (Figure 8B): Os(II) coordination partially localizes the π-system of the arene while protecting the coordinated double bond, enabling selective hydrogenation of the diene portion even under ambient conditions [53–54]. Reduction in the presence of a simple Pd/C catalyst yields desirable cycloalkenes after oxidative decomplexation in excellent yield. The same reaction with D_2_ produces a single isomer in which all the deuterium atoms are *anti* to osmium.

Earlier authoritative reviews by Harman provide comprehensive accounts of the rich chemistry accessible through dihapto-coordinating dearomatization agents, and the reader is directed to these for a detailed overview of the reaction classes achieved [45–47]. Nonetheless, a number of recent contributions based on the economically more sustainable W(0)- and Mo(0)-derived complexes have emerged that capture, with particular elegance, the synthetic power and versatility of this approach – selected examples that merit closer examination here.

**Scheme 2:**
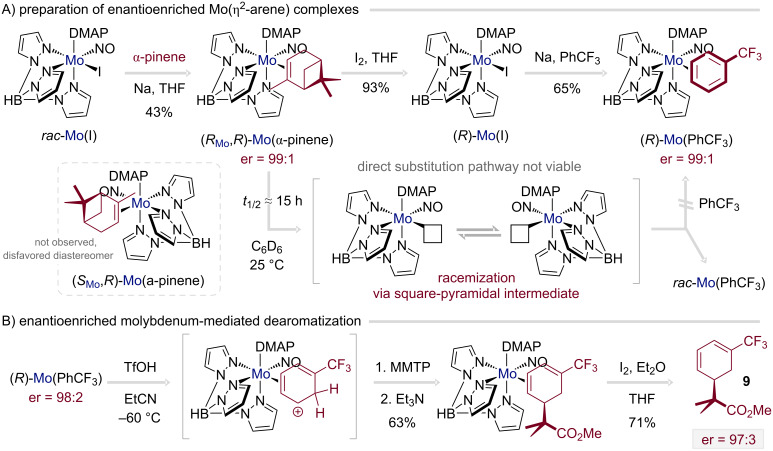
Synthesis and utilization of enantioenrichted Mo(η^2^-arene) complexes in enantioselective synthesis [55].

A key development is the realization of enantioenriched dearomatization employing a Mo(0) platform [55], extending a strategy previously confined to chiraly resolved Re(I) and W(0) systems [56–58]. Beyond the conceptual advance, the Mo(0) manifold offers practical benefits in terms of scalability and recyclability [59]. However, the preparation of enantioenriched η^2^-arene complexes presented unique challenges. In contrast to the Re(I) and W(0) analogues, where resolution of the metal center with α-pinene and subsequent ligand substitution proceeds with retention of configuration, the corresponding Mo(0) complex undergoes racemization during substitution (Scheme 2A). This obstacle was overcome by a redox-based approach: oxidation of the Mo(0)–α-pinene complex with iodine to a Mo(I) species, followed by reduction in trifluorotoluene, afforded the enantioenriched trifluorotoluene complex with essentially complete retention at the metal stereocenter (er = 99:1). However, control of the metal stereocenter alone is insufficient: a defined interaction with the prochiral aromatic ligand is necessary to set the absolute stereochemistry of the organic product. This relative stereochemistry depends on which face of the arene is coordinated and can be governed by high chiral recognition for binding (coordination diastereomer ratio, cdr) or stereoelectronic differentiation during subsequent electrophilic addition. Although the trifluorotoluene complex exists as a mixture of its two coordination diastereomers, the pronounced electronic asymmetry of the molybdenum fragment channels protonation to a single η^2^-arenium intermediate. This enables nucleophilic addition of a masked enolate (1-methoxy-2-methyl-1-(trimethylsiloxy)propene, MMTP) and subsequent oxidative demetalation to furnish the enantioenriched diene **9** (er = 97:3; Scheme 2B).

**Scheme 3:**
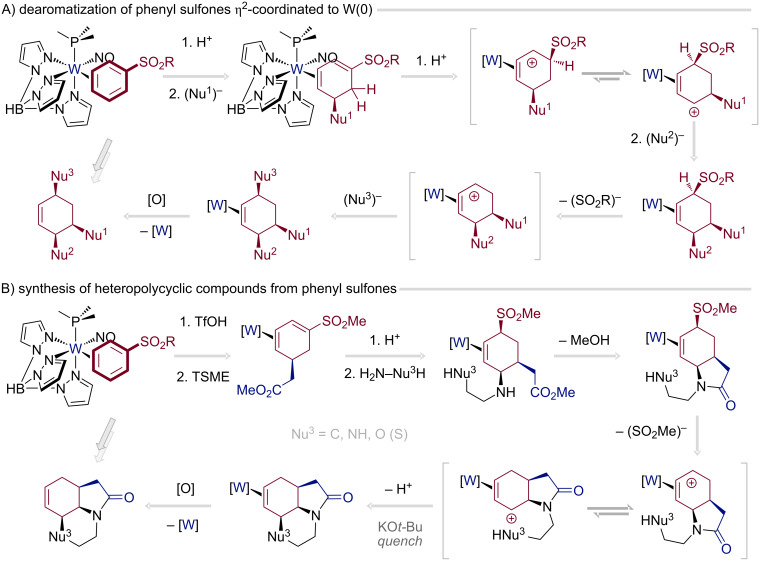
Synthesis of trisubstituted cyclohexenes from phenyl sulfones enabled by tungsten-mediated dearomatization; TSME: 1-(*tert*-butyl-dimethylsilyloxy)-1-methoxyethene [60,62].

Beyond the economic appeal of second-row Mo(0) systems, the TpW(NO)(PMe_3_) fragment remains the most versatile dihapto-coordinating dearomatization platform, combining pronounced π-basicity with practical scalability. Exploiting these attributes, a new multistep dearomatization protocol has recently been developed, enabling the conversion of arenes into trisubstituted cyclohexenes through three discrete nucleophilic additions to an η^2^-phenyl sulfone complex of W(0) (Scheme 3A) [60]. As established previously [61], protonation of η^2^-arene ligands bearing electron-withdrawing substituents generates reactive arenium intermediates that react with nucleophiles to furnish disubstituted η^2^-cyclohexadiene complexes. A second protonation/nucleophilic addition sequence can then proceed within the coordination sphere to deliver trisubstituted cyclohexenes. When applied to phenyl sulfones, this sequence markedly broadened the accessible chemical space, as substitution of the sulfonyl group by a third nucleophile enabled the synthesis of a diverse array of cyclohexene derivatives beyond sulfone and sulfonamide motifs.

By combining ester enolate and amine addition sequences, this methodology was subsequently extended to the synthesis of architecturally complex polyheterocyclic frameworks (Scheme 3B) [62]. Such scaffolds are scarcely represented in contemporary chemical literature, underscoring the potential of this strategy as a powerful platform for molecular discovery and medicinal chemistry. A similar strategic approach was utilized by Harman and co-workers to transform η^2^-coordinated anisole into a library of 3,6-substituted cyclohexenes with distinct biological relevance [63].

**Scheme 4:**
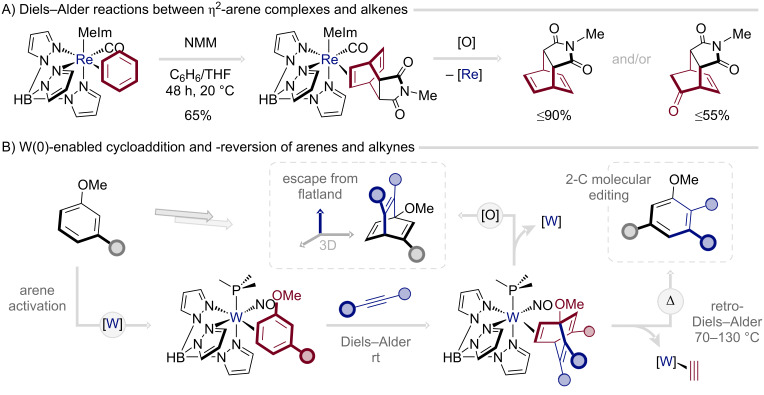
Diels–Alder reactions of η^2^-arene complexes with alkenes and alkynes; NMM: *N*-methylmaleimide [64–65].

A final notable advance lies in the deployment of the W(0) dearomatization platform to mediate Diels–Alder reactions of η^2^-coordinated benzenes with alkynes, affording the corresponding η^2^-barrelene complexes under ambient conditions [64]. While Diels–Alder cycloadditions between η^2^-arene complexes and alkenes are well-established (Scheme 4A) [65–66], analogous transformations with alkyne dienophiles had remained elusive. Oxidative liberation of the resulting complexes furnished several unprecedented free barrelenes (Scheme 4B). Intriguingly, η^2^-coordination of barrelenes to the W(0) center was also found to promote the *retro*-Diels–Alder process, effecting the formal extrusion of acetylene and generating modified arenes. This transformation thus represents both a rare example of a Diels–Alder reaction of benzenes and a remarkable two-carbon molecular editing of the aromatic framework.

Collectively, η^2^-coordination has established itself as a conceptually rich and versatile strategy for arene activation via π-bond localization. The recent development of structurally and electronically diverse η^2^-arene complexes has significantly broadened the synthetic landscape, enabling transformations that were previously inaccessible through conventional methods. Beyond their mechanistic elegance, these systems have demonstrated tangible utility in constructing complex molecular architectures with high precision. However, a key obstacle to the broader use of η^2^-coordinating dearomatization agents in synthesis is their reliance on stoichiometric quantities of the activating metal. Although recycling strategies offer partial relief, true progress depends on achieving catalysis through transient arene complexation. Unlike the well-established η^6^-coordination mode [67–68], η^2^-binding remains untapped in catalysis, with only a few isolated examples of redox-mediated ligand substitution hinting at its feasibility [47].

### η^3^-Benzyl complexes as intermediates in dearomative redox catalysis

#### Binding and structure

**Scheme 5:**
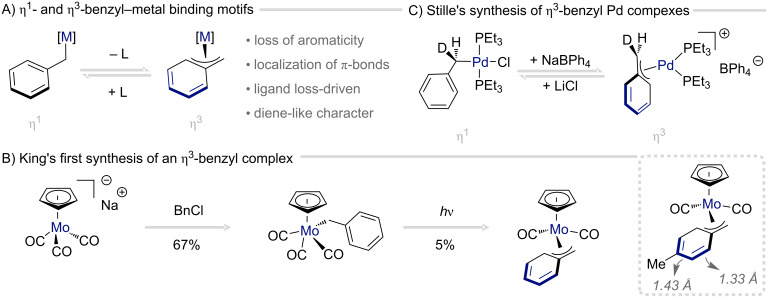
Binding characteristics and pioneering examples of isolable η^3^-benzyl complexes.

Within the landscape of redox catalysis, η^3^-benzyl complexes have emerged as rare yet remarkably versatile intermediates in synthetic methodology, offering unique opportunities for the development of dearomatization chemistry. In analogy to dihapto coordination (see previous section), the selective engagement of a transition metal with one C–C double bond of an arene π-system induces localization within the aromatic ring, resulting in a residual diene motif, a phenomenon reflected in the alternating C–C bond lengths of the ring [69]. Over the past decades, this coordination mode has been explored across a spectrum of transition metals, with palladium and nickel serving as the principal platforms. Distinct from their η^3^-allyl counterparts, which readily isomerize between σ (η^1^) and π (η^3^) coordination, the η^3^-benzyl motif demands partial disruption of aromatic stabilization – an energetic penalty that must be compensated by the metal–ligand interaction (Scheme 5A). This challenge can be addressed by the generation of a vacant coordination site through ligand abstraction, enabling effective π-binding. A landmark contribution by King and Fronzaglia in 1966 (Scheme 5B) elegantly demonstrated this concept: photolytic removal of a carbonyl ligand from a molybdenum precursor yielded the first-of-its-kind η^3^-benzyl complex [70]. The resulting compound exhibited a striking upfield shift of the five aromatic proton resonances (2–5 ppm), and its C(3)–C(7) bond lengths alternated between 142 pm and 133 pm – hallmarks of π-bond localization within the arene [71–72].

Another notable early example was reported by Stille and co-worker in 1978, who elegantly demonstrated the formation of an η^3^-benzyl–palladium complex via oxidative addition of Pd(0) to a benzyl halide (Scheme 5C) [73]. Detailed stereochemical and NMR analyses revealed a temperature- and solvent-dependent equilibrium between η^1^- and η^3^-coordination modes, proceeding with an impressive 94% net retention of configuration at the benzylic carbon.

#### Key intermediates in dearomative catalysis

**Figure 9:**
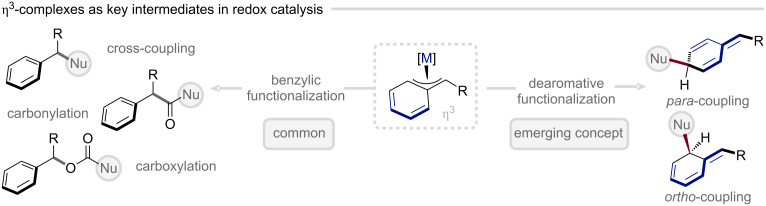
Divergent functionalization of benzyl electrophiles leveraging η^3^-benzyl complexes toward benzylic vs. dearomative functionalization.

Building on this seminal discovery, considerable effort has since been directed toward the synthesis and structural elucidation of η^3^-benzyl complexes, as well as their mechanistic role in catalysis. Intriguingly, despite the inherent potential of the η^3^-benzyl motif to engage in dearomatization chemistry, most studies have focused on exploiting its reactivity at the “intuitive” benzylic position rather than harnessing its transiently disrupted aromaticity. This rich body of work, comprehensively reviewed by Trost and co-workers, will therefore not be the focus of the present discussion [69]. The following section will instead highlight how η^3^-benzyl intermediates can serve as strategic entry points for the construction of architecturally complex, three-dimensional molecular frameworks from simple arene precursors (Figure 9).

**Scheme 6:**
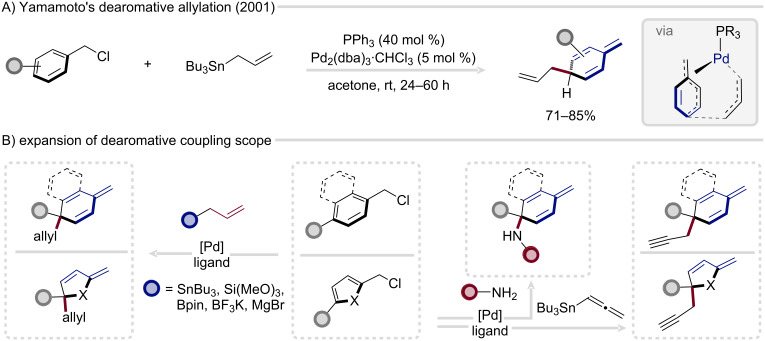
*p*-Selective allylation of benzyl chlorides with allylstannanes and subsequent synthetic expansion of dearomative cross-coupling.

A major conceptual milestone was achieved by Yamamoto and co-workers in 2001, who unveiled the first dearomatization of benzyl chlorides with allyltributylstannane under Stille coupling conditions (Scheme 6A) [74]. This elegant transformation demonstrated that transient disruption of aromaticity could be harnessed productively within a catalytic manifold. The authors proposed that a rearrangement of the η^3^-benzyl ligand from an *exo*- to an *endo*-coordination mode was pivotal in enabling the dearomatization process. Subsequent computational studies, however, refined this mechanistic understanding, revealing that reductive elimination proceeds through coupling of the C(3) terminus of the η^1^-allyl fragment with the *para*-carbon of the η^3^-benzyl ligand (Scheme 6A, right) [75]. Together, these insights established a mechanistic foundation for exploiting η^3^-benzyl intermediates as conduits for controlled arene-to-cyclohexadiene conversion.

Building on this foundational discovery, Bao and Yamamoto subsequently broadened the nucleophile scope to encompass allylsilanes [76], allylboronic acid derivatives [77], allylgrignards [78], amines [79], and allenylstannanes [80], thereby accessing the corresponding propargyl-functionalized products (Scheme 6B). In parallel, Bao and co-workers demonstrated that the dearomatization manifold extends beyond simple benzenoid systems to include heteroarenes such as furans, thiophenes, and pyrroles (Scheme 6B) [81–82]. Despite this diversification, the underlying reactivity pattern remained remarkably consistent, with preferential formation of 1,4-substituted dearomatized products observed across the substrate classes.

**Figure 10:**
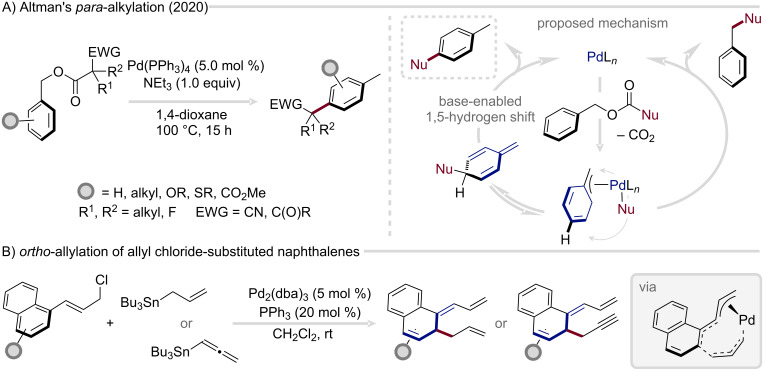
Strategies for *para*- and *ortho*-selective arene functionalization/dearomatization via η^3^-benzyl complexes.

A refined approach to controlling regioselectivity was introduced by Cheong and Altman in 2020 (Figure 10A) [83]. In this study, the authors explored an intramolecular, decarboxylative C–H functionalization of benzylic electrophiles, uncovering a decisive role of the base in governing site selectivity. Computational and mechanistic analyses revealed that only sufficiently strong, minimally hindered amine bases, such as triethylamine or quinuclidine, favored formation of the 1,4-substituted dearomatized product over simple benzylation. This outcome was rationalized by the involvement of the base in facilitating the 1,5-hydrogen transfer during the rearomatization step, thereby steering the reaction along the *para*-functionalization pathway.

A distinct and less frequently encountered pathway enables *ortho*-functionalization of arenes via intermediate η^3^-benzyl complex formation. Yamamoto and co-workers first showcased this in 2008 (Figure 10B), demonstrating the transformation of allyl chloride-substituted naphthalenes and phenanthrenes in the selective coupling with allylstannanes to deliver the corresponding *ortho*-allylated products [84]. Subsequently, Bao and co-workers extended this concept to encompass allenylstannanes, thereby accessing the propargylated analogues [80]. Computational investigations of this latter transformation identified an η^3^-allylnaphthalene–η^1^-allenyl palladium complex as the key to achieving *ortho*-selectivity (Figure 10B) [85–86].

**Scheme 7:**
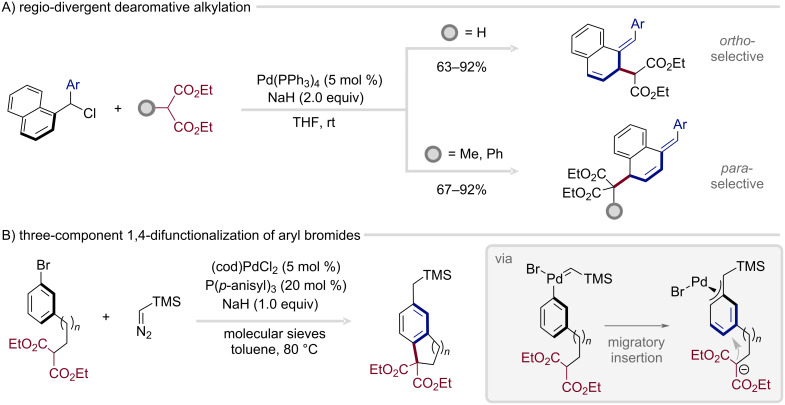
Substrate-dependent *ortho*- and *para*-selective dearomatization of naphthyl chlorides and leveraging three-component strategies to access η^3^-benzyl intermediates.

Building on these insights, the Bao group employed malonates as alkylating agents in reactions with (chloromethyl)naphthalene derivatives (Scheme 7A), thereby expanding the synthetic utility of the overarching platform [87]. This transformation furnished either *ortho*- or *para*-substituted carbocycles, depending on the substitution pattern of the malonate. Crucially, selective arene functionalization over benzylation required the presence of a phenyl substituent at the benzylic position. The nature of the malonate proved decisive: secondary malonates delivered the *ortho*-products, whereas tertiary malonates favored *para*-substitution. In a subsequent study, the same group introduced a ligand-controlled variant of this protocol, in which sterically demanding ligands promoted *para*-selectivity, while less encumbered systems channeled the reaction toward *ortho*-functionalization [88].

Following a conceptually related strategy, Yamaguchi and co-workers leveraged a three-component strategy that accesses the key η^3^-benzyl intermediates from aryl halides in combination with diazo compounds (Scheme 7B) [89–90]. Oxidative addition of the halide to palladium is followed by carbene formation, and facile migratory insertion to generate the η^3^-benzyl complex. In analogy to Bao’s protocol, this intermediate then undergoes selective nucleophilic attack by malonates. Both intra- and intermolecular versions of this reaction have been reported [91–94]. Notably, this three-component approach has recently been rendered enantioselective for the first time, marking a pivotal advance toward the construction of complex, three-dimensional molecular scaffolds from simple arene feedstocks and underscoring the vast, still largely untapped potential for methodological innovation in this arena [95].

### Activation of η^4^-coordinated arenes

**Figure 11:**
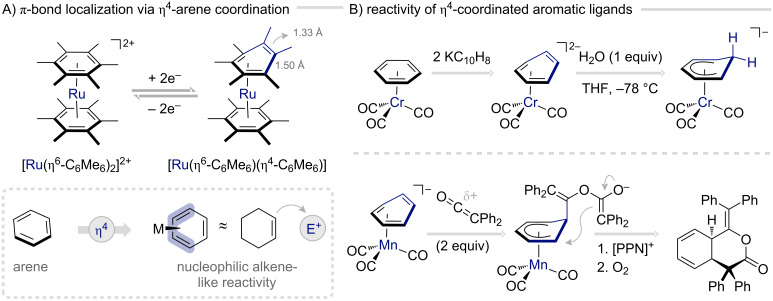
η^4^-Arene coordination as an underexplored but promising pathway for arene activation [96,98–100].

Among the less explored pathways in arene activation, η^4^-coordination of aromatic systems to electron-rich transition metal fragments stands out as a fertile yet underexplored frontier in dearomatization chemistry. By engaging two adjacent π-bonds, this interaction elegantly transforms a delocalized aromatic framework into an alkene-like substrate poised for selective reactivity. A canonical illustration is provided by the two-electron reduction of the symmetric sandwich complex [Ru(η^6^-C_6_Me_6_)_2_]^2+^, which furnishes the neutral [Ru(η^6^-C_6_Me_6_)(η^4^-C_6_Me_6_)] species upon haptotropic rearrangement, in accordance with the 18-electron rule (Figure 11A) [96–97]. Through partial coordination, the metal fragment localizes a subset of the π-system, effectively unmasking the latent double-bond character of the aromatic ring.

This is strikingly captured in the solid-state structure of the complex. One ring retains near-planarity, with a mean C–C distance of 1.41 Å, which is slightly elongated relative to free hexamethylbenzene (1.39 Å), consistent with pronounced metal-to-ligand backbonding. In contrast, the second ring departs from planarity altogether: two carbons tilt out of plane by 43° relative to the original plane. This deformation, induced by the addition of two electrons, profoundly disrupts π-bond delocalization, culminating in a C–C distance of 1.33 Å that corroborates a localized double-bond character.

Although the structural features of η^4^-coordinated arenes clearly indicate enhanced reactivity and access to alkene-like transformations, the seminal Ru system remains synthetically unexploited. However, pioneering studies of Cooper and co-workers have demonstrated that η^4^-coordination of the isoelectronic {Cr(CO)_3_}^2−^ and {Mn(CO)_3_}^−^ fragments can effectively unravel the latent reactivity of aromatic ligands, enabling a diverse array of organic transformations (Figure 11B) [99–100]. Beyond the η^6^-mode, characteristic of transition metal carbonyl chemistry, these electron-rich anions can engage arenes such as benzene and naphthalene in an η^4^-fashion, localizing their π-systems. In direct analogy to the ruthenium complex, two-electron reduction of the parent η^6^-arene complexes [Cr(CO)_3_(η^6^-C_6_H_6_)] and [Mn(CO)_3_(η^6^-C_6_H_6_)]^+^ induces ring slippage to furnish the η^4^-bound [Cr(CO)_3_(η^4^-C_6_H_6_)]^2−^ and [Mn(CO)_3_(η^4^-C_6_H_6_)]^−^ species.

As observed for η^2^-binding dearomatization agents, η^4^-coordination profoundly reshapes the intrinsic reactivity of the arene: the localized π-system now behaves as a nucleophilic alkene, displaying pronounced susceptibility toward electrophiles – an inversion of the conventional reactivity expected from transition metal carbonyl systems. Strikingly, even simple arenes such as benzene or naphthalene, when η^4^-bound to these highly basic metal fragments, can be protonated by mild acids to form thermally robust arenium complexes (Figure 11B). This remarkable basicity underscores the exceptional electron-donating power of the {Cr(CO)_3_}^2−^ and {Mn(CO)_3_}^−^ units, which stabilize dearomatized, cationic intermediates without auxiliary tethers.

Beyond protonation and deuteration, η^4^-arene complexes exhibit a compelling repertoire of C–C bond-forming reactions [101–103]. Notably, reactions with benzyl halides [99], iminium salts [104], nitrones [105], and ketenes [98] reveal rich synthetic potential. In a striking example, the [Mn(CO)_3_(η^4^-C_6_H_6_)]^−^ complex acts as a nucleophile toward diphenylketene to generate a cyclohexadienyl intermediate that captures a second ketene molecule (Figure 11B). Subsequent ring closure furnishes a product complex that, upon oxidation with molecular oxygen, liberates the [2 + 2 + 2] bis-adduct, dihydroisochroman-3-one, in 73% yield. This sequence elegantly illustrates how η^4^-coordination can orchestrate multistep bond construction. Finally, η^4^-bound intermediates have been proposed as intermediates in catalytic transformations – most notably in Chirik’s molybdenum-catalyzed arene hydrogenations [106–107] – highlighting the emerging potential of this activation mode in catalysis.

## Conclusion and Future Directions

Arenes remain privileged scaffolds in synthesis, recognized for their abundance and structural diversity. The deliberate perturbation of aromaticity through π-bond localization has emerged as a powerful strategy for molecular complexity generation. Coordination of transition metals to defined arene π-fragments provides a particularly elegant means of selective activation, yet the widespread adoption of this paradigm has been limited by the scarcity of predictable, catalytic variants. Among the established platforms discussed in this review, η^3^-benzyl complexes stand out as catalytically competent intermediates in redox-catalytic transformations. Future advances in this arena will depend on broadening the nucleophile portfolio, exerting precise catalyst control over site-selectivity (spanning benzylic, *ortho*, and *para* manifolds) and unlocking enantioselective variants to access three-dimensional, chiral architectures. In contrast, η^2^- and η^4^-coordination modes have been explored extensively from a structural and stoichiometric reactivity standpoint, yet catalytic manifestations remain conspicuously rare. The energetic cost of aromaticity loss, compounded by the strong binding of dearomatized intermediates, presents the key barrier toward catalysis. Overcoming this challenge will require new conceptual leaps – for instance, the design of catalytic processes that proceed under retention of aromaticity, thereby enriching the repertoire of arene functionalization beyond the established cross-coupling and C–H activation canon. Alternatively, merging arene activation with additional concepts, such as photoredox catalysis offers an exciting opportunity to address thermodynamic constraints and to harness light-driven redox events for selective, efficient arene diversification.

## Data Availability

Data sharing is not applicable as no new data was generated or analyzed in this study.
